# A longitudinal study on the occurrence of *Cryptosporidium *and *Giardia *in dogs during their first year of life

**DOI:** 10.1186/1751-0147-49-22

**Published:** 2007-09-11

**Authors:** Inger S Hamnes, Bjørn K Gjerde, Lucy J Robertson

**Affiliations:** 1 Norwegian School of Veterinary Science, Department of Food Safety and Infection Biology, Section of Microbiology, Immunology and Parasitology, P.O. Box 8146 Dep, N-0033 Oslo, Norway; 2 National Veterinary Institute, Section for Parasitology, P.O. Box 8156 Dep., N-0033 Oslo, Norway

## Abstract

**Background:**

The primary aim of this study was to obtain more knowledge about the occurrence of *Cryptosporidium *and *Giardia *in young dogs in Norway.

The occurrence of these parasites was investigated in a longitudinal study by repeated faecal sampling of dogs between 1 and 12 months of age (litter samples and individual samples). The dogs were privately owned and from four large breeds. Individual faecal samples were collected from 290 dogs from 57 litters when the dogs were approximately 3, 4, 6, and 12 months old. In addition, pooled samples were collected from 43 of the litters, and from 42 of the mother bitches, when the puppies were approximately 1 and/or 2 months old.

**Methods:**

The samples were purified by sucrose gradient flotation concentration and examined by immunofluorescent staining.

**Results:**

128 (44.1%) of the young dogs had one or more *Cryptosporidium *positive samples, whilst 60 (20.7%) dogs had one or more *Giardia *positive samples. The prevalence of the parasites varied with age. For *Cryptosporidium*, the individual prevalence was between 5.1% and 22.5%, with the highest level in dogs < 6 months old, and declining with age. For *Giardia*, the individual prevalence was between 6.0% and 11.4%, with the highest level in dogs > 6 months old, but the differences between age groups were not statistically significant. Significant differences in prevalences were found in relation to geographic location of the dogs. Both parasites occurred at low prevalences in Northern Norway.

**Conclusion:**

Both *Cryptosporidium *and *Giardia *are common in Norwegian dogs, with *Cryptosporidium *more prevalent than *Giardia*. Prevalences of the parasites were found to be influenced by age, geographical location, and infection status before weaning.

## Background

*Giardia *and *Cryptosporidium *are intestinal protozoan parasites of animals and humans, causing asymptomatic to severe intestinal infections, depending on the virulence of the *Cryptosporidium *or *Giardia *isolate involved and the immunological capabilities of the hosts. *Cryptosporidium *infections are common in humans and calves, but also occur in dogs, cats, pigs, horses, sheep, goats and wildlife [1]. *Giardia *infections are common in humans and livestock, but also occur frequently in dogs, cats and numerous species of wild mammals and birds [2]. Studies on the prevalence of *Giardia *and *Cryptosporidium *in other animal species in Norway have shown a prevalence of 49% and 12%, respectively, in dairy calves between 0–6 months of age [3]. Among wild cervids (moose, reindeer, roe deer and red deer) the prevalence of *Cryptosporidium *was found to range between 0% and 6.2% in the different species, and the prevalence of *Giardia *was found to be between 1.7% and 15.5% in the different species [4]. In 684 litters of suckling piglets the prevalences were found to be 8.3% *Cryptosporidium *positive and 1.5% *Giardia *positive [5]. In Norwegian red fox (*Vulpes vulpes*), a *Cryptosporidium *prevalence of 2.2% and a *Giardia *prevalence of 4.8% were found [6].

Currently there are 14 commonly accepted species of *Cryptosporidium *[7-9]. Dogs can be naturally infected by *Cryptosporidium canis*, *C. parvum *and *C. meleagridis *[10,11]. *C. canis *infections in dogs are usually asymptomatic, but may cause severe diarrhoea, malabsortion and weight loss [12].

There are currently six recognized species of *Giardia*, but only *Giardia duodenalis *is known to infect multiple host species, including humans [13,14]. Molecular genetic studies have demonstrated that *G. duodenalis *is a species complex comprising at least 7 major genotypes/assemblages [15]. Most of these assemblages appear to have distinct host associations. Genotyping of *Giardia *isolates from dogs has shown that *Giardia *from Assemblages A, B, C and D may occur in this host [16-18]. Traub et al. (2005) [19] found genetically identical isolates in a dog and two humans in the same household, indicating zoonotic transmission between humans and dogs. The majority of *Giardia *infections in dogs are asymptomatic, but some infected dogs may suffer from acute or chronic diarrhoea, weight loss, poor weight gain despite a normal appetite, and, less commonly, vomiting and lethargy [20]. The Parasitology laboratory at the Norwegian School of Veterinary Science (NVH) has sporadically diagnosed both *Cryptosporidium *and *Giardia *from several domestic species, including dogs. The aim of this study was to obtain more knowledge about the occurrence of these two parasite genera among young Norwegian dogs.

## Methods

### Material

Norwegian breeders of Labrador Retrievers, Newfoundland Dogs, Leonbergers and Irish Wolfhounds had been recruited by the Department of Companion Animal Clinical Sciences, at NVH, to participate in a large clinical study regarding associations between dog breed, growth rate, nutrition, and skeletal disease, from birth until 24 months of age [21-23]. The dogs in the present study were a sub-set of more than 600 dogs participating in the clinical investigation outlined above, and were monitored from approximately one month of age until about 12 months of age.

The overall sampling period was between November 1999 and July 2002. The breeders were asked to provide a faecal sample from the bitch and a pooled sample from the litter when the puppies were about 1 and 2 months old, and to recruit the new owners of the puppies to participate in the study (the dogs were delivered to their new owners at approximately 8 weeks old). The new owners of the puppies were asked to collect a faecal sample from their dogs when the dogs were approximately 3, 4, 6, and 12 months old. The samples were sent to the Parasitology Lab at the NVH, Oslo, with information about date of sampling, dog identity (name, date of birth, breed) and the name and address of the owner. The samples were kept refrigerated from arrival until processing at the lab. For each dog, only one sample was included in each age group. If two samples from the same dog were provided within an age group, the sample that was collected when the dog was closest to the "ideal age" in the group (i.e. 3-, 4-, 6- or 12-month-old) was included in the study and the other sample excluded. When dogs were found to be *Giardia-*positive the owners were recommended to treat their dogs with fenbendazole (50 mg/kg on 3 consecutive days).

In total, the material consisted of 1–4 faecal samples from each of 290 individual pure-bred, privately-owned, household dogs originating from 57 different litters, giving a total of 887 samples. There were 142 male dogs, 147 female dogs and one dog with unknown sex. Each litter consisted of 1–11 puppies (mean 5.1, median 5). In addition there were a total of 75 pooled-samples from 43 different litters and a total of 69 individual samples from 41 different mother bitches. The ages of the mother bitches were between 27 and 93 months. Seven breeders participated with more than one litter during the course of the study, 6 with 2 litters (total of between 8 and 16 dogs), and one breeder participated with dogs from 3 litters (total of 4 dogs).

Some of the owners/breeders missed one or more requested sampling occasions for unknown reasons. Thus the number of samples included in the different age groups differs from the overall number of participating dogs, litters or bitches. The actual numbers on which the calculations were based are given in the Tables or in the text.

For some analyses the dogs were divided into 3 groups: originating from a litter with negative samples; originating from a litter that had been found positive for *Cryptosporidium *and/or *Giardia*; or originating from a litter with unknown status (i.e. that had not been sampled when still in the litter, but only as individual dogs).

### Sample analysis

The faecal samples were analyzed by a sucrose flotation concentration and immunofluorescent staining method as described by Olson et al. (1997) [24], and modified as described by Hamnes et al. (2006) [3]. Briefly; each pooled sample was thoroughly mixed, then a small amount of faeces (average weight, 2.9 g) was suspended in approximately 10 ml of phosphate buffered saline solution (PBS; 0.9% NaCl, pH 7.2) and mixed to a homogenous suspension. The suspension was then filtered through a surgical gauze sponge to yield approximately 7 ml of filtrate. The filtrate was layered on top of 5 ml of 1 M sucrose (specific gravity 1.13) for clarification and centrifuged at 800 × g for five minutes to concentrate the cysts/oocysts. The interface and the upper layer of liquid were carefully collected with a pipette and transferred to a clean tube and recentrifuged (800 × g; 5 min). The supernatant was decanted and the pellet resuspended in PBS to a volume of 1 ml. Thirty μl volumes of the suspension were air-dried to microscope slides, methanol fixed and stained with fluorescein-labelled (FITC) monoclonal antibody to oocysts of *C. parvum *and cysts of *G. duodenalis *(A100FR FLR Aqua-Glo from Waterborne Inc, New Orleans, USA). After incubation, excess antibody was washed off and the slides air-dried before mounting (DABCO/glycerol mounting medium 2%) with a 22 × 22 mm cover slip. The area under the cover slip was examined using an epifluorescent microscope (Leica DMLB) at 200× and 400× magnification, using an I3 filter with blue excitation and band pass filter (BP) 450 – 490 nm. With each batch of stained slides a known positive sample was stained and used as a control.

Samples were classified as negative (no cysts/oocysts found), or positive, the latter being graded as 1+ when < 5 cysts/oocysts on average were present in each of 20 fields of view, as 2+ with 5 to 10 cysts/oocysts on average in each of 20 fields of view, or as 3+ with > 10 cysts/oocysts on average in each of 20 fields at 400× magnification, respectively. The size of some of the cysts/oocysts was measured with a calibrated eyepiece graticule to ensure that they were within the size range given for *Cryptosporidium *and *Giardia*. A portion of the original faecal sample was also examined by a standard egg counting technique for helminth eggs and *Isospora *oocysts.

### Detection level of method

Ten 3-gram faecal samples were seeded with either 100, 1000 or 5000 cysts/oocysts per gram faeces and processed according to the method described above. For *Giardia*, 10/10 samples were found positive in all 3 seeding categories. For *Cryptosporidium*, 7/10 samples seeded with 100 oocysts per gram were found positive, and 10/10 samples seeded with either 1000 or 5000 oocysts were found positive. Thus, this method has a detection level of 100 *Giardia *cysts and at least 1000 *Cryptosporidium *oocysts per gram when 3 grams of faecal material are examined.

### Statistical analyses

Statistical tests included χ^2 ^and Fishers' Exact test for analyzing 2 × 2 contingency tables, odds ratio calculations, t-test for comparisons of means, and confidence interval calculations. Differences were considered statistically significant if p < 0.05.

The prevalences of *Cryptosporidium *and/or *Giardia *were evaluated with respect to age, intensity of infection, infection status before weaning (positive/negative/unknown), geographical distribution (in which part of Norway the dog was raised), number of samples provided from each dog, sex, seasonal differences, and multiple parasitic infections.

## Results

### Litters and bitches

Of the 40 litters examined at one month of age, only one litter (2.5%) was found to be *Cryptosporidium *positive, whereas 2 litters were *Giardia *positive (5.0%). Of the 39 bitches sampled at the same time as their puppies, none were *Cryptosporidium *positive. One bitch was *Giardia *positive (2.6%), but her litter was not *Giardia *positive on that occasion.

Of the 35 litters examined at two months of age, eight (22.9%) were positive for *Cryptosporidium*, whereas none was *Giardia *positive. Of the 29 bitches sampled at this time, one (3.4%) was *Cryptosporidium *positive, and her litter was also *Cryptosporidium *positive at that time. None of the bitches were positive for *Giardia *at this sampling.

### Individual dogs

Prevalences of the two parasites in each age group, including the litters, are given in Table 1.

**Table 1:** Prevalences of *Cryptosporidium *and *Giardia *in dogs in different age groups

**Age category (months)**	**Total number of samples**	***Cryptosporidium *positive samples**		***Giardia *positive samples**	
		
		**Number (%)**	**95% CI**	**Number (%)**	**95% CI**
1^a^	40	1 (2.5)	<0.01–14.2	2 (5.0)	0.6–17.5
2^a^	35	8 (22.9)	11.9–39.4	0 (0.0)	0.0–11.9
3	264	57 (21.6)^b^	17.1–27.0	23 (8.7)	5.8–12.8
4	249	56 (22.5)^c^	17.7–28.1	15 (6.0)	3.6–9.8
6	216	28 (13.0)^b,c,d^	9.1–18.2	17 (7.9)	4.9–12.3
12	158	8 (5.1)^b,c,d^	2.5–9.9	18 (11.4)	7.3–17.4

^a ^Pooled samples from litter

^b,c,d^, Variables with the same superscript are significantly different from each other. Differences were considered statistically significant if p < 0.05.

**Figure 1:**
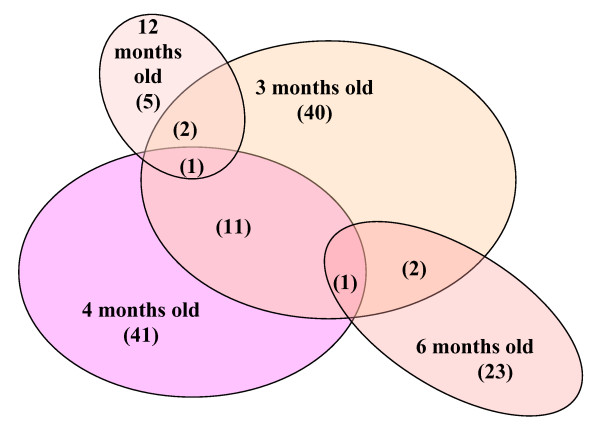
***Cryptosporidium *positive dogs**. Diagram showing the number of dogs that were positive for *Cryptosporidium *at 3, 4, 6 and 12 months of age, or at more than one sampling.

Of the total of 887 samples from 290 individual dogs, 149 (16.8%) were positive for *Cryptosporidium*. One hundred and twenty-eight (44.1%) of the dogs had one or more *Cryptosporidium *positive samples (109, 17, and 2 dogs had 1, 2, or 3 positive samples respectively) during the study (Figure 1).

**Figure 2:**
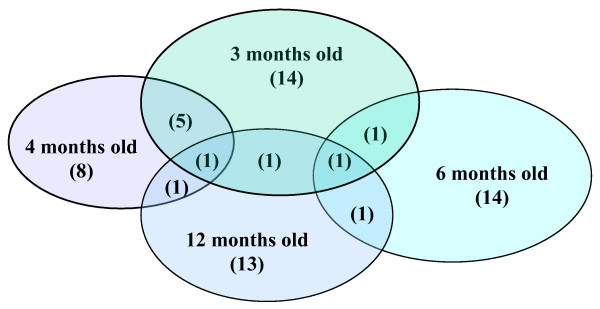
***Giardia *positive dogs**. Diagram showing the number of dogs that were positive for *Giardia *at 3, 4, 6 and 12 months of age, or at more than one sampling.

**Figure 3:**
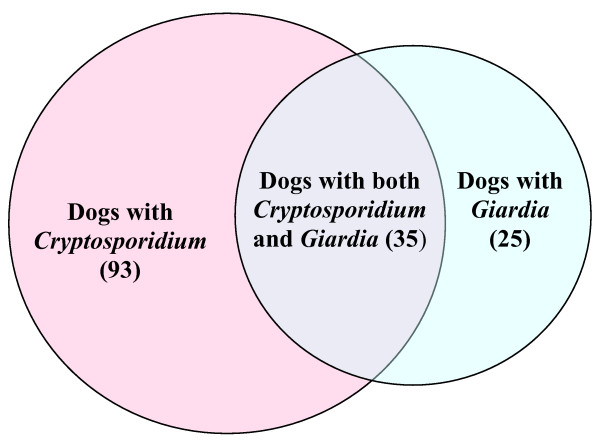
**Occurrence of *Cryptosporidium *and/or *Giardia *among the positive dogs**. Diagram showing the number of dogs that were positive for *Cryptosporidium *and/or *Giardia *during the study.

Seventy-three (8.2%) of the 887 samples were positive for *Giardia*, and 60 (20.7%) dogs had one or more *Giardia *positive samples (49, 9, and 2 dogs had 1, 2, or 3 positive samples respectively) during the study (Figure 2). Of the 290 dogs, 153 (52.7%) had one or more samples with *Cryptosporidium *and/or *Giardia *(Figure 3) during the study.

### Age differences – intensity of infection

*Cryptosporidium *was most prevalent among the youngest dogs with the prevalence declining with age (Tables 1 and 2). Individual dogs in the 3- and 4-month-old groups had significantly higher prevalences of *Cryptosporidium *than the older dogs (p-values between 0.02 and < 0.0001). Dogs in the 6-month-old group had a significantly higher prevalence of *Cryptosporidium *than dogs in the 12-month-old group (p = 0.01). There were no significant differences in the prevalence of *Giardia *when comparing the four age groups. Among the 226 dogs that were sampled at both 3 and 4 months, 26.5% of the 3-month-old *Cryptosporidium *positive dogs were also positive at 4 months. For *Giardia*, 31.6% of the 3-month-old *Giardia *positive dogs were also positive at 4 months. Cumulative prevalence and percentage of new positives for both parasites in the different age groups are given in Table 2.

**Table 2:** Cumulative prevalence of *Cryptosporidium *and *Giardia *and percentage of new positives in different age groups of dogs

**Age category (months)**	***Cryptosporidium *Cumulative prevalence^a ^in %**	***Giardia *Cumulative prevalence^a ^in %**	***Cryptosporidium***		***Giardia***	
			
			**Number of dogs**	**New positives in %**	**Number of dogs**	**New positives in %**
**3**	19.7	7.9	264	21.6	264	8.7
**4**	34.5	11.0	177^b^	20.1	228^b^	3.1
**6**	42.3	16.2	121^b^	17.4	171^b^	5.8
**12**	44.1	20.7	77^b^	6.4	114^b^	9.6

^a ^based on 290 participating dogs.

^b ^dogs found negative in (all) preceding age groups; positive dogs were excluded from the following age group(s).

Results on level of intensity of infection related to age are given in Table 3. Within the different age groups there were no significant differences between the mean age of the positive and negative animals, or between the positive ones for the two parasites.

**Table 3:** Intensity of infection with *Cryptosporidium *and *Giardia *in dogs related to average age. All samples from individual dogs.

	**Intensity of infection^a^**				**Intensity of infection^a^**			
	
	***Giardia *0**	***Giarda *1+ **(% of pos. samples)	***Giardia *2+ **(% of pos. samples)	***Giardia *3+ **(% of pos. samples)	***Crypto *0**	***Crypto *1+ **(% of pos. samples)	***Crypto *2+ **(% of pos. samples)	***Crypto *3+ **(% of pos. samples)
**Number of samples**	814	19 (26.0)	17 (23.3)	37 (50.7)	738	102 (68.5)	22 (14.8)	25 (16.7)
**Average age (days)**	176^a,b^	228^a^	185	178	185^c,d^	135^b,c^	154	136^d^
**Dual infections**	0	2	2	11	0	11	2	2

^a ^as described in 'Material and methods'.

Variables with the same superscript are significantly different from each other

### Geographical distribution

Among the litters, the majority was born in Eastern (58%) and Western Norway (21%), but the puppies were sold to owners all over the country, with a high degree of geographical dispersal of dogs from the different litters; for instance, the 18 dogs living in Oslo County originated from 13 different litters. For the whole country (19 counties), the average number of dogs from each litter represented in a county was 1.5 (1.0–3.3 dogs). When comparing the prevalences in the different regions and counties with each other (a dog being classified as positive if the actual parasite was identified in the dog during the course of the study), dogs living in Northern Norway had the lowest prevalences of both parasites (Table 4). There was a significantly higher percentage of *Cryptosporidium *positive individuals among dogs in Eastern Norway compared with dogs in Northern Norway (p = 0.0063, OR = 3.61) or Western Norway (p = 0.0271, OR = 1.97), as well as in dogs in Mid Norway compared with dogs in Northern Norway (p = 0.0145, OR = 4.29).

**Table 4:** Prevalences of *Cryptosporidium *and *Giardia *in dogs related to the geographical location of the participating dogs

**Geographical region**	**Eastern Norway**	**Southern Norway**	**Western Norway**	**Mid Norway**	**Northern Norway**	**Total**
No. dogs/No. samples	116/353	39/129	76/218	26/84	29/91	286^a^/875
No. *Cryptosporidium *positive samples (%)	72 (20.4) ^b^	19 (14.7)	30 (13.8)	19 (22.6) ^c^	7 (7.7) ^b,c^	147 (16.8)
No. *Cryptosporidium *positive dogs (%)	62 (53.5) ^d,e^	14 (35.9)	28 (36.8) ^d^	15 (57.7) ^f^	7 (24.1) ^e,f^	126 (44.1)
No *Giardia *positive samples (%)	35 (9.9) ^g^	14 (10.9) ^h^	18 (8.3) ^i^	5 (6.0)	1 (1.1) ^g,h,i^	73 (8.3)
No. *Giardia *positive dogs (%)	28 (24.1) ^j^	11 (28.2)^k^	15 (19.7)	5 (19.2)	1 (3.5) ^j,k^	60 (21.0)

^a ^Four dogs moved during the study and were excluded from these calculations;

^b-k ^– variables with the same superscript are significantly different from each other

For *Giardia *there was a significantly higher percentage of *Giardia *positive dogs among dogs from Eastern Norway compared with dogs from Northern Norway (p = 0.0096, OR = 8.91), and in dogs from Southern Norway compared with dogs from Northern Norway (p = 0.0094, OR = 11.00). Significantly fewer dogs from Northern Norway were *Giardia *positive than were dogs from other parts of the country (p = 0.0139, OR = 0.12).

### Seasonal differences

There appeared to be a tendency towards higher prevalences of both parasites in winter, but no definite conclusions about this could be made due to the clustered nature of the data.

### Number of samples provided from each dog

The likelihood of a dog being detected as positive increased with increasing number of samples examined. Thus, dogs from which only one sample was examined had a significantly lower prevalence of *Cryptosporidium *than dogs represented by more than one sample. For *Giardia*, the only significant difference was found between 2 and 4 samples, but there was a substantially higher prevalence of *Giardia *in dogs represented by 3 or 4 samples than in those with fewer samples (Table 5).

**Table 5:** Prevalence of *Cryptosporidium *and *Giardia *related to number of samples from each dog

**Number of samples provided from each dog**	**Total number Of dogs**	**Number of *Cryptosporidium *positive dogs (%)**	**Number of *Giardia *positive dogs (%)**
**1**	43	8 (18.6)	5 (11.6)
**2**	35	14 (40.0)	3 (8.6)
**3**	74	40 (54.1)	17 (23.0)
**4**	138	66 (47.8)	35 (25.4)
**Total**	**290**	**128 (44.1)**	**60 (20.7)**

A dog was considered positive if it had at least one positive sample with *Cryptosporidium *or *Giardia*. Some of the dogs had more than one positive sample for either or both parasites.

### Infection status before weaning

The dogs were divided into 3 different groups according to whether they came from a litter that had tested positive for *Cryptosporidium *and/or *Giardia *at 1 or 2 months of age, from a litter that was negative at 1 and 2 months of age, or from a litter with unknown litter status (litters not sampled). Comparing these groups revealed that dogs from positive litters and dogs with unknown litter status had a significantly higher prevalence of *Cryptosporidium *at 3 months of age than dogs from negative litters (p = 0.04, OR = 2.32 and p = 0.01, OR = 2.61, respectively). No significant differences were found in the other age groups, or for *Giardia *in any of the four age groups.

### Sex

No significant differences in the prevalences of *Cryptosporidium *and *Giardia *were found between male and female dogs. Among the female dogs 45.5% were *Cryptosporidium *positive at some point in the study, whereas 43.7% of the male dogs were *Cryptosporidium *positive. For *Giardia*, 22.1% of the female and 19.7% of the male dogs were *Giardia *positive at some point in the study.

### Multiple parasitic infections

Thirty-five dogs were positive for both parasites during the study, either at the same sampling (15 dogs) or at different samplings (data not shown). Twenty-five (27.3%) of the 128 *Cryptosporidium *positive dogs were also positive for *Giardia *at some point, whereas 25 (15.4%) of the 162 *Cryptosporidium *negative dogs, were *Giardia *positive. This difference was statistically significant (p = 0.0190, OR 2.06). Eighty-seven percent of these dogs were *Cryptosporidium *positive before or concurrent with their *Giardia *infection(s). Among these dogs, 31.4% had *Cryptosporidium *and/or *Giardia *at more than one sampling (i.e., 2 or 3 positive samples), whereas among the dogs being diagnosed with only one parasite, 13.6% were found positive more than once. This difference was significant (p = 0.0223, OR = 2.92). Eight (22.8%) of the 35 dogs that were positive for both *Cryptosporidium *and *Giardia *were also diagnosed with ≥100 eggs per gram faeces (EPG) of other intestinal parasites (*Toxocara canis *and/or *Toxascaris leonina*) 1–3 times during the study (data not shown), whereas 11 (9.3%) dogs with either *Cryptosporidium *or *Giardia*, and 10 (7.3%) dogs negative for *Cryptosporidium *or *Giardia *had ≥100 EPG of nematode eggs. The differences in prevalence of nematode infections between the *Cryptosporidium *and *Giardia *positive dogs and the two other groups were statistically significant (p < 0.05).

## Discussion

The prevalence of *Giardia *in individual dogs ranged between 6.9% and 11.4% in the different age groups examined, and the *Cryptosporidium *prevalence ranged between 5.1% and 22.5%. This is within the range reported in other studies. Thus, the prevalence of *Giardia *in dogs has been found to be between 5.4% and 55.2% [25-32], whereas the prevalence of *Cryptosporidium *has been reported to range from 0% to 44.8% [33,26,29,36,31].

The prevalences of both *Cryptosporidium *and *Giardia *are variable in different hosts and within the same host species, and depend on a number of factors including age, living conditions, diagnostic methodology and region studied. Other factors that also might influence the prevalences are season, purebred/mixed bred, feeding, urban/rural living conditions, single or multiple household dogs, treatment, and immune status. These variables must be kept in mind when comparing the results from different studies, as well as the fact that the dogs in this study originated from only 57 litters. Thus, the littermates might have shared a common infection source (kennel/breeder) that might have affected the results at 3 and possibly 4 months old.

It is interesting, but not surprising, that the occurrence of *Cryptosporidium *among the dogs at 3 months old was found to be associated with infection status of the litter before weaning. In addition to the known positive litters, several other litters/individual dogs had apparently become infected with *Cryptosporidium *and/or *Giardia *between litter sampling at 2 months of age and individual sampling at 3 months, both among litters with unknown status and among previously negative litters. The higher *Cryptosporidium *prevalence among dogs from positive litters at 3 months old may suggest that it takes some time for the puppies to rid themselves of the infection, which is consistent with the findings of Lloyd and Smith (1997) [37], that some dogs may shed *Cryptosporidium *oocysts for more than 80 days. They also found that the oocysts shedding was intermittent with several peaks in all the 6 participating dogs, and 5 out 6 dogs shed oocysts for more than 60 days after becoming infected.

Several dogs in the current study were infected with the parasites at more than one sampling. This may be due to chronic infections or re-infection. The dogs that had one or more negative samples in between the positive samples may either have been re-infected or might have had a continuous infection with intermittent shedding of cysts/oocysts or shedding below the detection limit of the method used. One would also expect that the stress associated with weaning and moving to a new environment may have compromised the immune system of the puppies and made them more susceptible to infection or less able to rid themselves of an already existing infection, thus contributing to the high prevalences in the youngest dogs. Since genotyping of *Cryptosporidium *and *Giardia *was not performed during this study, it is not possible to determine whether the dogs diagnosed with either of these parasites more than once had a persistent infection with the same species (*C. canis*/*C. parvum*) or genotype (*Giardia duodenalis *assemblages A, B, C, D) or whether they had been re-infected with another species/genotype in between samplings. Autoinfection is known to occur for *Cryptosporidium *and reinfection with *Giardia *is also common. Little is known about the extent to which acquired immunity after an infection with a particular *Cryptosporidium *species or *Giardia duodenalis *genotype will protect against infection with another species or genotype. Findings in cattle [38-41] have shown that different *Cryptosporidium *species predominate in different age groups. This may suggest that an infection with *C. parvum *does not provide immunity against *C. bovis*, *C. andersoni *and *Cryptosporidium *deer-like genotype. *Giardia *is known to induce poor immunity in the host and re-infections frequently occur, as documented in cattle [42].

Many dogs were found to be both *Cryptosporidium *and *Giardia *positive during the course of the study, which is to be expected due to the similar epidemiology of these parasitic infections and the repeated sampling regimen used in the current study. It may, however, also be suggested that some dogs are more susceptible to parasitic infections than others. In the present study, dogs that were positive for both *Cryptosporidium *and *Giardia *also had a significantly higher prevalence of helminth infections than dogs that were negative for one or both parasites. It is also possible that some of the dogs lived in a contaminated environment with a high possibility of becoming (re)infected.

The results herein show that the prevalences of *Cryptosporidium *varied significantly with the age of the dogs, but also with the number of samples examined from each animal (Tables 1, 2 and 5), this is as expected, as increasing the sampling frequency obviously increases the possibility of detecting an infection. The shedding of *Giardia *cysts is known to be intermittent and the general recommendations for diagnosis is examination of 3 samples collected during a limited time span (i.e. from a day to a week) to enhance the chances of detecting infection. *Cryptosporidium *might also be shed intermittently in dogs [30], so that a single sample testing regimen (one sample from each dog in each age category) as used in this study is likely to underestimate the prevalence of both parasites, but in particular *Giardia*. The prevalence remained high in the 3- and 4-months-old group and then declined. *Cryptosporidium *has been reported to occur commonly in dogs less than six months of age, whereas adult dogs are less frequently infected [43].

For *Giardia *the highest prevalence was found among dogs in the 12-months-old group, but differences in the prevalence between the different age groups were not significant. As the dogs became older, the percentage of new positives increased (Table 2). Cross-sectional studies have shown *Giardia *to be most prevalent among dogs less than 6 months of age [28,35]. However, Huber et al. (2005) [35] examined only 35 dogs of less than 6 months of age. Kirkpatrick (1988) [31] examined faecal samples from 2294 dogs presented to a veterinary teaching hospital, and found the highest *Giardia *prevalence in dogs less than 2 years old. Fontanarrosa et al. (2006) [28] found that the prevalence of *Giardia *was higher in pure-breed dogs than in mixed-breed dogs. The prevalence of *Giardia *in the current study also varied with the dog age and the number of samples examined from each dog, but not to the same extent as for *Cryptosporidium*.

The mother bitches were not positive for *Giardia *or *Cryptosporidium *at the same time as their puppies, except for one concurrent *Cryptosporidium *infection of a bitch and her litter. However, due to the lack of genotyping data it is impossible to determine whether the bitches and litters were infected with the same species/genotype or whether the infections were unrelated.

The prevalences of both parasites were found to be higher in winter than in spring and summer, but this finding must be interpreted with caution due to the (clustered) nature of the data in the present study. There may be a tendency towards a higher prevalence of the parasites in winter, but this tendency cannot be separated from the effect that several positive dogs from one or more litters would have had on the results within a season, and one cannot say with any degree of certainty that the differences were truly related to season. Several other studies have found seasonal differences in the prevalence of *Giardia *in dogs [44,27,28,31,17], but Nolan and Smith (1995) [45] did not.

There were significant differences in the prevalence of both parasites between different regions in Norway. These differences might be due to demographic patterns and variations in density of dogs. Eastern Norway, which had the highest prevalences of both parasites, also had the highest number of dogs (2.4) per km^2^, whereas Northern Norway had the lowest density with 0.4 dogs/km^2 ^[46,47]. Moreover, the low prevalences of both parasites in Northern Norway may be related to a mostly rural demographic pattern in combination with harsh climatic conditions, which offers less opportunities for dog-to-dog contact (directly or indirectly) and thus a reduced probability of exposure to infective cysts/oocysts. Kirkpatrick (1988) [31] found that an urban locality gave a higher risk of parasitic infection compared with a non-urban locality. Since *G. duodenalis *of Assemblage A occurs in a wide range of mammalian hosts, including humans, livestock, wild animals and pets, such as cats [13], dogs might also become infected with *Giardia *from sources other than dogs. Climatic differences might also influence the prevalence of the parasites in the different areas. Northern Norway usually has long cold winters. Studies by Robertson and Gjerde (2004, 2006) [15,48] suggested that *Giardia *and *Cryptosporidium *have only limited survival in the environment under Norwegian winter conditions.

There were differences in the intensity of infection related to age for both *Giardia *and *Cryptosporidium*. *Cryptosporidium *positive dogs were on average younger than *Cryptosporidium *negative dogs, consistent with previous knowledge about *Cryptosporidium *and *Giardia *infections in dogs. *Giardia *is more frequently found in preadult and adult dogs, and shedding of *Giardia *cysts can last for months, whereas *Cryptosporidium *oocyst shedding usually only lasts a few weeks [49]. Interestingly, 50.7% of the *Giardia *positive dogs had the highest level of intensity of infection (3+), whereas only 16.7% of the *Cryptosporidium *positive dogs were in the same category. *Giardia *is often considered to cause a 'chronic' infection, with a long period of low cyst excretion, so therefore one would have expected a high number of samples with low cyst numbers. Possibly, the duration of peak shedding for *Giardia *in dogs is substantially longer (~5 weeks) than for *Cryptosporidium *(1–2 weeks), as found in calves [49]. Moreover, the peak intensity of *Cryptosporidium *oocyst excretion may have occurred before the individual sampling of the dogs commenced, when they were about 3 months old.

Owners of *Giardia *positive dogs were recommended to treat their dogs with fenbendazole, which is one of several treatment options for *Giardia *infections in dogs, and this might have reduced the *Giardia *prevalence. However, data on whether the owners actually treated their dogs were not collected and despite this treatment recommendation, some of the dogs had *Giardia *in more than one sample. This could either be re-infection(s) or persistent infections. Fenbendazole is reported to have good effect against *Giardia *infections in dogs [50-52]. However, the time intervals between the samplings in the current studies (1, 2 and 6 months) were sufficient for the dogs to be re-infected between sampling occasions. Many of the efficacy studies performed on fenbendazole treatment of giardiasis in dogs have only followed the dogs for 3 days up to 4 weeks after treatment and have reported treatment efficiency to range between variable to good [50,53,52]. Decock et al. (2003) [51] evaluated 4 different treatments against canine giardiasis and found that 18 days post treatment, all but one of the dogs in the different groups were positive again. Metronidazole gave the best results; all 6 dogs were negative on day 10 post treatment, but by day 18 they were all shedding cysts again. Both Decock et al. [51] and Beelitz et al. [50] reported re-infection after treatment (within 18 and 28 days post treatment) with different compounds and treatment regimens. The long term effects of treatment on giardiasis status are unknown. O'Handley et al. (2000) [42] found that calves treated against *Giardia *with fenbendazole were reinfected within 2 weeks after treatment, and that this pattern of reinfection was consistent after every treatment period.

Since genotyping of *Giardia *and *Cryptosporidium *had not been established at our laboratory at the time of the investigation, information about which *Cryptosporidium *species/genotypes and *Giardia *species/assemblages were present in these samples is lacking. More recently, our laboratory has identified *Cryptosporidium cani*s from three *Cryptosporidium *positive dog samples. Five *Giardia *positive samples from dogs have also been genotyped; 3 were closely related to *G. duodenalis *specific host dog (Assemblage C) and 2 were *G. duodenalis *Assemblage B (unpublished data).

## Conclusion

Both *Cryptosporidium *and *Giardia *are common in Norwegian dogs, with *Cryptosporidium *being more prevalent than *Giardia*. Since *Cryptosporidium canis *from dogs can infect humans, and dogs can harbour *Giardia duodenalis *of the zoonotic genotypes of Assemblages A and B, further studies with genotyping of isolates of these parasites from Norwegian dogs are necessary to evaluate their public health significance in Norway.

## Competing interests

The author(s) declare that they have no competing interests.

## Authors' contributions

All three authors were involved in the planning of the study. ISH performed the faecal exams, performed the statistical analyses, drafting and revising of the manuscript. BKG and LJR have been involved in drafting and critical revision of the manuscript. All authors have approved the manuscript.
